# Assessing river discharge dynamics through relative surface water extent changes in river basins

**DOI:** 10.1016/j.isci.2024.111598

**Published:** 2024-12-14

**Authors:** Feng Mao, Margaret Shanafield, Val Ouellet, David M. Hannah, Stefan Krause

**Affiliations:** 1 Institute for Global Sustainable Development, School for Cross-faculty Studies, University of Warwick, Coventry CV4 7AL, UK; 2 College of Science and Engineering, Flinders University, Tonsley 5042 SA, Australia; 3 Atlantic Salmon Federation, St. Andrews, NB E5B 3S8, Canada; 4 School of Geography, Earth and Environmental Sciences, University of Birmingham, Birmingham B15 2TT, UK

**Keywords:** Earth sciences, Methods in earth sciences, Physical geography, Hydrology

## Abstract

Surface water in rivers is vital for human society. However, our current understanding of the dynamics and drivers of river flows relies predominantly on stream gauging data, which are limited in spatial coverage and involve significant costs. Remote sensing techniques have emerged as complementary tools for monitoring river discharge, but these satellite-based methods often require complex data processing. This paper introduces a simple, yet effective and robust methodology to understand river discharge, using Australian rivers as a case study. Our findings reveal that changes in relative surface water extent within river basins can effectively capture river discharge dynamics. Moreover, we observe a linear relationship between relative surface water extent and river discharge in hilly basins and a quadratic relationship in flat basins. The new approach helps monitor river flows in ungauged river basins, and it has the potential to bridge the gap between local and regional understandings of water dynamics.

## Highlights

• Changes in surface water extent capture river discharge dynamics effectively
• Water extent and discharge show a linear relationship in hilly basins
• Water extent and discharge show a quadratic relationship in flat basins
• Study aids understanding of river discharge in ungauged basins

## Graphical abstract

**Figure:**
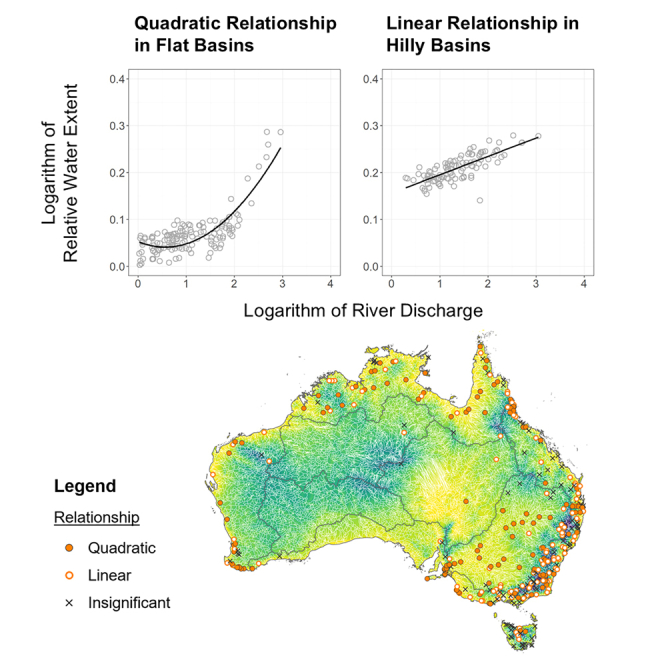

## Introduction

Surface water in rivers is fundamental to the functioning of ecosystems and the security of human society, offering essential resources for drinking, irrigation, and industrial activities. In the Anthropocene, river systems face significant pressures from climate change and human water usage. Our primary understanding of river flow regimes largely relies on the analysis of stream gauging data. However, due to their high financial and maintenance cost, stream gauges are often sparse and not uniformly distributed across river systems, inadequately capturing the spatial variability of river flows.^1^ Furthermore, gauges require regular maintenance and calibration, which becomes increasingly challenging in remote and inaccessible areas. Therefore, there is a clear need for alternative methods to enhance our understanding of the evolution of river flow dynamics across space and time.

Previous research has demonstrated the feasibility and efficacy of remote sensing for estimating river discharge.^2^^,^^3^^,^^4^^,^^5^ These approaches generally fall into three categories. The first category includes methods that rely on width-based or water-surface-elevation-based measurements alone. For instance, the At-Many-stations Hydraulic Geometry (AMHG) method and its derivatives, Bayesian AMHG-Manning (BAM), estimate discharge from satellite-derived river width measurements.^3^^,^^6^^,^^7^ In parallel, water-surface-elevation-based approaches such as space-based rating curves use satellite altimetry to relate water level to discharge,^8^^,^^9^ and quantile mapping techniques further refine these relationships through statistical models.^10^^,^^11^ These approaches are suitable for understanding broad and generalized relationships between river discharge and channel morphology, rather than capturing non-linear dynamics ranging from regional to local scales. The second category combines river width and water surface elevation with hydraulic characteristics (e.g., slope) to enhance discharge estimation. These approaches typically utilize Manning’s equation and leverage data from satellite missions such as the Surface Water and Ocean Topography (SWOT) for estimating discharge.^12^^,^^13^ This strategy is particularly useful in complex environments where more detailed and integrated hydraulic information is available. The third strategy involves comparing remotely sensed land surfaces within and outside river channels, known as C/M (calibration/measurement) methods. For example, Brakenridge et al.^14^ used the Advanced Microwave Scanning Radiometer (AMSR-E) band to assess brightness temperature in reference and inundation areas, with their ratio reflecting river discharge levels. Tarpanelli et al.^15^ demonstrated the use of optical sensors such as Sentinel-3 and MODIS to infer river discharge. Since this strategy does not require local hydraulic information, it is applicable for areas without high-resolution spatial data or with smaller rivers and sparse vegetation coverage.^2^^,^^16^

**Figure 1:**
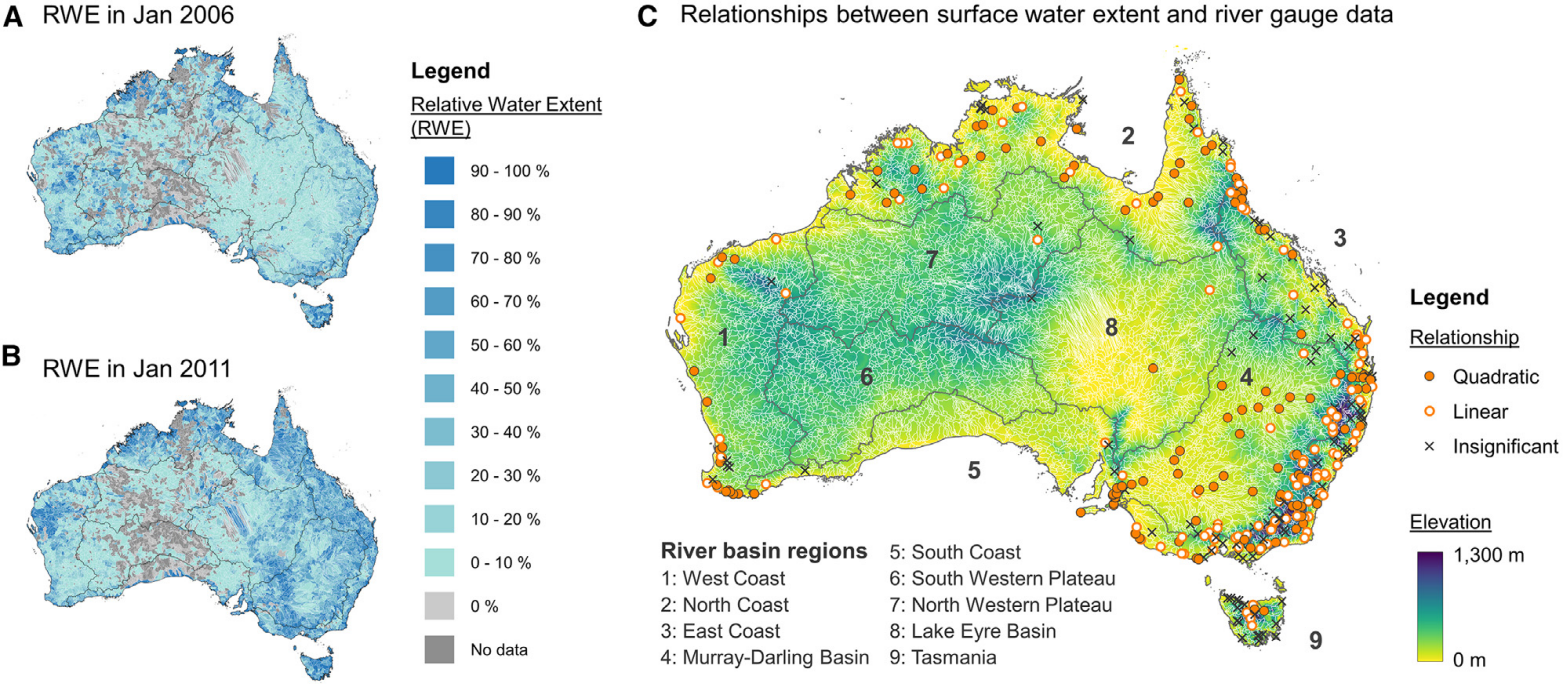
Relative water extent (RWE) in Australia (A and B) RWE for two example months showing its temporal dynamics: (A) January 2006 and (B) January 2011 and (C) relationships between RWE and river gauge data. Dark gray lines denote the boundaries of nine river basin regions in Australia (level 3 PFAF basins), and thin white lines delineate the level 8 basin boundaries.

Nevertheless, these existing methodologies rely on generalizable relationships that are often violated when water resource management alters natural flow relationships. These methods necessitate intricate data processing and substantial computational efforts that can be daunting for many specialists and limits the accessibility of remote sensing techniques for deriving river discharge information. To overcome these limitations, this study introduces an alternative methodology that leverages regional, remotely sensed data of spatial surface water extents to capture river discharge dynamics. We demonstrate this simple, yet effective, and robust approach using Australian rivers as a case study (Figure 1). We investigate the relationship between spatial surface water extents in river basins and river discharge measurements at river gauges and examine how this relationship is affected by various factors. Furthermore, we explore how the proposed new approach can enhance our understanding and management of water resources and risks.

## Results

### Three types of relationship

**Figure 2:**
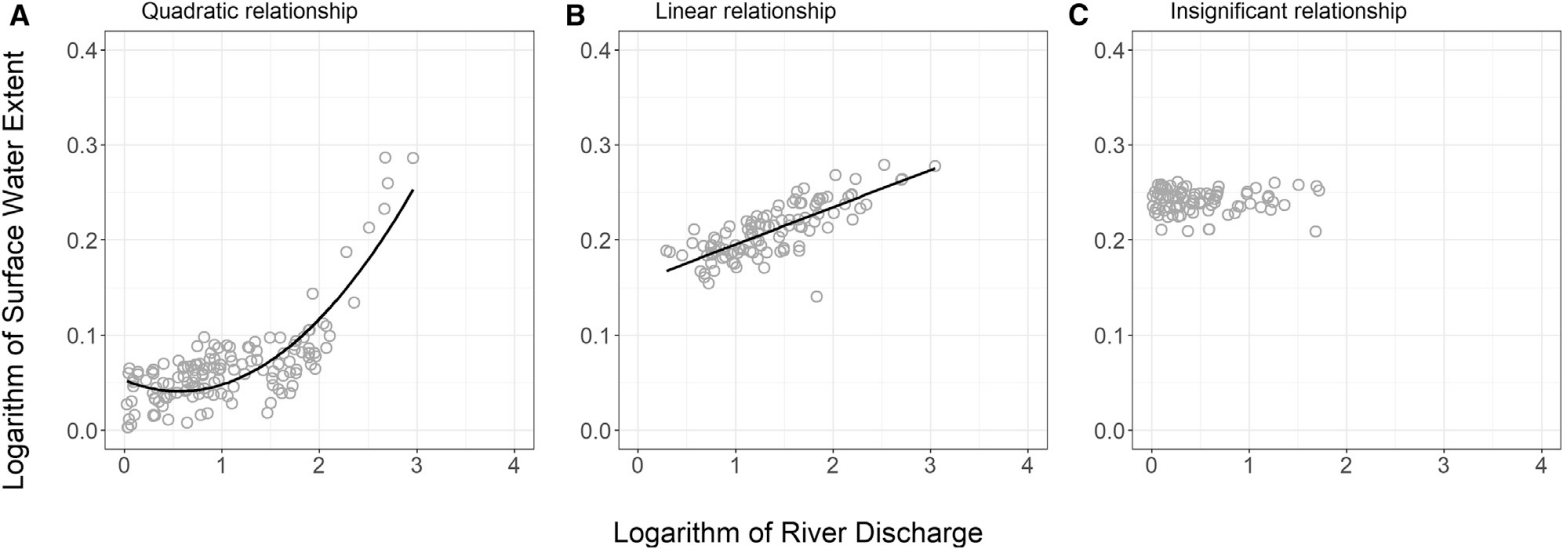
Examples of the three relation types (A) A quadratic relationship (R^2^ = 0.6855; the quadratic coefficient’s *p* < 0.001) between river discharge from the station GRDC5204450 and relative water extent from the basin PFAF56463030. (B) A linear relationship (R^2^ = 0.5906; *p* < 0.001) between river discharge from the station GRDC5101161 and surface water extent from the basin PFAF56310061. These relationships were considered significant because the two-sided probability (*p*) values for the corresponding coefficients were less than 0.05. (C) An insignificant relationship between river discharge from the station GRDC5202055 and surface water extent from the basin PFAF56391106.

The relationships between the river-gauging-based discharge data at 346 stations and the remote-sensing-based data of RWE in their basins were tested (Figure 1C). Among the basins, 108 had an insignificant relationship and 238 had a significant relationship, in which 113 and 125 were linear and quadratic relationships, respectively (see examples in Figure 2).

The 346 GRDC stations were distributed across 298 PFAF basins, in which 39 basins had more than one station (up to five stations per basin). In these multi-station basins, a high degree of consistency in the relationships between Q and RWE was observed. Specifically, the models concurred on the significance of the relationships in 31 basins and agreed on the type of relationship (quadratic, linear, or insignificant) in 24 basins.

### Influencing factors

Two variables were included in the selected binary logistic regression model for predicting the significance of the relationship between river discharge and surface water extent. A higher number of months with available data (B > 0, *p* < 0.001) and a lower mean slope of the river basin (B < 0, *p* < 0.001) were associated with an increased likelihood of observing a significant relationship between Q and RWE (Table 1). The model demonstrated an overall correct prediction rate of 77.2% and was considered a good fit to the data, as indicated by the non-significant Hosmer-Lemeshow test result (*p* = 0.058), suggesting no significant deviation between the predicted and observed probabilities, and the pseudo R-squared value (Nagelkerke R-square = 0.305).

**Table 1:** Results of the binary logistic regression analysis for testing which variables predicted a significant or insignificant relationship between river discharge and surface water extent

Variable	B	S.E.	Wald	df	Sig.	Exp(B)	95% C.I. for EXP(B)	
							Lower	Upper
Number of months with data	0.021	0.003	48.949	1	<0.001	1.021	1.015	1.027
Mean slope	−0.160	0.030	28.183	1	<0.001	0.852	0.803	0.904
Constant	0.083	0.258	0.103	1	0.748	1.086	–	–

Dependent variable encoding: Insignificant = 0; Significant = 1. *N* = 346. B stands for the coefficient for the independent variable. S.E. denotes standard error associated with B. Exp(B) refers to the exponentiation of B, which is also known as the odds ratio.

A further binary logistic regression analysis was performed on the station-basin pairs that exhibited a significant relationship. This analysis identified the variables predictive of whether the relationship was linear or quadratic. Three variables were included in the selected model, two of which had a significant effect (*p* < 0.05). Specifically, a lower mean slope of the river basin (B < 0, *p* = 0.011) and a higher percentage of scattered tree cover (B > 0, *p* = 0.005) were indicative of a greater likelihood for a quadratic relationship between Q and RWE (Table 2). Scattered trees had a very high odds ratio and a wide 95% confidence interval, suggesting that this variable had a strong influence on the relationship. The selected model had an overall correct prediction rate of 60.5%, which was a moderate fit to the data, according to the Hosmer-Lemeshow test (*p* = 0.396) and the pseudo R-squared value (Nagelkerke R-square = 0.151).

**Table 2:** Results of the binary logistic regression analysis for testing which variables predicted the linear or quadratic relationship between river discharge and surface water extent

Variable	B	S.E.	Wald	df	Sig.	Exp(B)	95% C.I. for EXP(B)	
							Lower	Upper
Mean slope	−0.085	0.034	6.395	1	0.011	0.918	0.860	0.981
Open hummock grasses	−7.239	3.963	3.337	1	0.068	0.001	0.000	1.695
Scattered trees	9.851	3.493	7.954	1	0.005	1.898×10^4^	20.187	1.784×10^7^
Constant	0.376	0.241	2.446	1	0.118	1.457	–	–

Dependent variable encoding: Linear = 0; Quadratic = 1. *N* = 238. B stands for the coefficient for the independent variable. S.E. denotes standard error associated with B. Exp(B) refers to the exponentiation of B, which is also known as the odds ratio.

## Discussion

### Relationship between river discharge and surface water extent

The results of this study reveal that the dynamics of river discharge in the investigated catchments and conditions can be captured to a large degree by the observed changes in surface water extent. However, the strength and accuracy of the relationship between Q and RWE are influenced by the availability of monitoring data describing their correlation and the environmental characteristics of river basins. These findings open new opportunities for alternative approaches to surface water monitoring: the established relationship between Q and RWE can be used to construct an understanding of water resources in ungauged river basins, indicating not only *where* the presence of surface water is but also allowing an estimation of *how much* water is discharged along a river, based on readily and freely available satellite imagery.

The number of months with data is one of the most critical variables determining whether a significant relationship can be detected (Table 1). It implies that this approach performs better in river basins for which sufficient observational data are available to develop a robust relationship between Q and RWE. This finding reiterates statements within the scientific community on the importance of long-term data for understanding and managing freshwater systems.^17^

The likelihood of detecting a significant relationship is reduced in river basins with steeper slopes (Table 1). This may be attributed to the fact that rivers in hilly basins, often characterized by a low stream order, may be too narrow to be effectively captured by satellite imagery. For instance, the spatial resolution of the Global Surface Water dataset is 30 m, which exceeds the width of many upstream headwater streams. The geology of Australia has been stable for a comparatively long time, resulting in relatively flat topography over much of the country^18^; therefore, it provided an ideal environment for the use of this method.

Of the different landcover variables that were tested in the analysis for their impact on the correlation between Q and RWE, none predicted the significance of their relationship (Table 1). The presence of vegetation shading was proven to be not critical for determining whether a significant relationship between Q and RWE can be detected. It suggests that the proposed approach is robust even in river basins with extensive forest coverage. With regard to topographic influences, the mean slope of a river basin was shown to be important for distinguishing between linear and quadratic relationships between Q and RWE (Table 2). Surface water extent and river discharge are more inclined toward a linear relationship in hilly river basins, whereas a quadratic relationship is more likely to be encountered in flatter regions (e.g., Murray-Darling Basin and North Coast basins in Figure 1). This distinction could stem from differing flooding mechanisms: in hilly basins, excess water is primarily channeled through river paths and valleys, whereas in flatter basins, the same volume of water tends to overflow riverbanks, inundating a larger area of the surrounding floodplain.

### A new remote sensing of discharge strategy

The proposed approach represents a new strategy that distinguishes itself from other existing methods for remote-sensing-based discharge estimation. Unlike other methods that rely for instance on comparing inundated areas to selected dry areas using passive microwave sensing data,^14^ our method quantifies the surface water extent at the river basin level without requiring local comparisons. The data requirements for our approach are significantly less complex than those of traditional hydraulic measurements, such as river surface width^3^^,^^7^ or water levels,^8^ eliminating the need to measure cross-sections along the river or to employ altimetry data in the analysis. The surface water extent data utilized here is readily available from existing hydrological data products and services,^19^^,^^20^ making it relatively straightforward to obtain or process, even with limited expertise in remote sensing.

Although this paper provides evidence that proves the advantages of the proposed approach, future research may further refine and expand upon it in several ways. First, conducting a sensitivity analysis on river basin boundaries could prove beneficial. Our study utilized level 8 basins from the HydroBASINS dataset, with considerations of computational efficiency and data availability (see STAR Methods). Where data permit, exploring river basins of varying scales or those delineated based on drainage areas contributing to a gauge could yield additional insights. Second, investigating the underlying mechanisms of the linear and quadratic relationships observed in various river basin types warrants further study. This would benefit from the inclusion of additional factors beyond land surface and cover variables. In turn, exploring how the classification of these relationships can provide new insights into understanding hydrological processes and managing water resources and risks would also be valuable. Lastly, there is significant potential for applying this approach in other regions worldwide, which may present different hydrological and environmental conditions, as well as greater data availability, thereby enhancing our understanding of its global applicability.

In addition to enhancing our understanding of the relationship between RWE and Q, as demonstrated earlier, there is considerable potential to advance this method beyond capturing river discharge dynamics, toward directly estimating discharge. To achieve this, further research is required to effectively identify the linear and quadratic relationships specific to each river basin type, ensuring the appropriate relationship is used to translate surface water information into discharge. Validation is needed to assess its accuracy (e.g., by comparing RWE-derived Q with gauge-measured Q) before confidently applying this discharge estimation method in ungauged or poorly gauged basins.

### Connecting local and regional understanding of water resources

The proposed methodology provides a powerful approach that enables to integrate local and regional understanding of water dynamics. Data gaps in areas without river gauges can be filled by remote sensing products providing information of surface water extent. Therefore, this methodology allows for scaling up from localized discharge monitoring to enhance regional water security insights.

In this study, we deliberately chose not to use the boundaries of the drainage basins that contribute their water to the GRDC river gauges. Instead, we applied HydroBASINS as a more universal river basin delineation system, which is independent of the locations of river gauges. The analysis (Relationship between river discharge and surface water extent) supports that correlations are likely between the dynamics of surface water extent in a basin and the discharge data from a river gauge within the basin, and the river gauge does not need to be at the outlet of the river basin.

This characteristic holds significant promise for research and practices concerning regional and large-scale water resources and security, particularly where detailed, local, in-channel data are of less importance. The findings of this study enable us to reliably interpret changes in relative surface water extent within basins as indicative of water quantity dynamics in the associated rivers, which aids the advancement of the understanding and management of regional water resources,^21^^,^^22^ scarcity,^23^^,^^24^^,^^25^ and flood risks.^26^^,^^27^^,^^28^

### Conclusions

This paper presents a new approach for capturing river discharge dynamics using satellite imagery, characterized by its simplicity, efficacy, and robustness. Stream discharge dynamics are represented based on remotely sensed surface water extent information, which does not require advanced remote sensing expertise for data processing and analysis since all relevant remote sensing data have been made available through GSWE and Google Earth Engine. A significant correlation between the relative surface water extent and river discharge, as recorded at river gauges, has been observed in more than two-thirds (238/346) of the station-basin pairs considered in this study. Furthermore, the robustness of this approach is underscored by its independence from factors like land cover and vegetation shading. Thus, this methodology provides an alternative strategy for understanding the dynamics of surface water resources, offering valuable insights particularly for interdisciplinary research communities and practitioners.

### Limitations of the study

This study proposes a method to capture river discharge dynamics through changes in relative surface water extent. However, further development is required to directly estimate river discharge from relative surface water extent data. Although the research has identified linear and quadratic relationships between river discharge and relative surface water extent across various river basin types, the underlying mechanisms require further investigation. Moreover, these relationships were established using the HydroBASINS dataset at a specific scale in Australia. Future work could explore these relationships across different spatial scales, drainage area delineations, and regions globally.

## Resource availability

### Lead contact

Requests for further information and resources should be directed to and will be fulfilled by the lead contact, Feng Mao (Feng.Mao@warwick.ac.uk).

### Materials availability

This study did not generate any new materials.

### Data and code availability

• This paper analyses existing publicly available data. The data sources are listed in the key resources table.
• This paper does not report original code.
• Any additional information required to reanalyze the data reported in this paper is available from the lead contact upon request.

## Acknowledgments

We acknowledge the support from the European Union’s Horizon 2020 Research and Innovation Programme under the Marie Skłodowska-Curie Grant Agreement No. 734317 (HiFreq) and the Cardiff University’s Darlithwyr Disglair Programme.

## Author contributions

Conceptualization, F.M., M.S., S.K.; methodology, F.M.; validation, M.S., V.O.; formal analysis, F.M.; visualization, F.M.; writing—original draft, F.M.; writing—review & editing, F.M., M.S., V.O., D.H., S.K.

## Declaration of interests

The authors declare no competing interests.

## Declaration of generative AI and AI-assisted technologies in the writing process

During the revision of this work, the authors used ChatGPT in order to improve readability. After using this tool/service, the authors reviewed and edited the content as needed and take full responsibility for the content of the publication.

## STAR★Methods

### Key resources table

**Table:** 

REAGENT or RESOURCE	SOURCE	IDENTIFIER
**Data**		

Global Surface Water Explorer (GSWE) dataset	Donchyts et al.^19^	https://global-surface-water.appspot.com/
River discharge	Global Runoff Data Center Data Portal	https://grdc.bafg.de/GRDC/EN/Home/homepage_node.html
Dynamic Land Cover Dataset (v2.1)	Geoscience Australia^29^	https://researchdata.edu.au/dynamic-land-cover-version-21/1278349
GEODATA 9 Second Digital Elevation Model (DEM-9S, Version 3)	Geoscience Australia^30^	https://researchdata.edu.au/geodata-9-second-grid-2008/1277764
Australian river basin boundaries	HydroBASINS	https://www.hydrosheds.org/products/hydrobasins

**Software**		

R	The R Project for Statistical Computing	https://www.r-project.org/
SPSS	IBM SPSS Statistics	https://www.ibm.com/products/spss-statistics

### Method details

#### Study area

As one of the largest countries globally, Australia provides a unique opportunity to test the proposed approach on a continental scale due to its extensive hydrological records. The country hosts a diverse range of rivers and habitats constrained by aridity and seasonality, with flow regimes ranging from perennial to highly ephemeral.^31^ Although many of the rivers and streams have been heavily altered over the past two centuries, there are also extensive river systems that flow naturally.^18^ Overall, Australian rivers host a high number of endemic fish species,^32^ although decreasing streamflows are changing aquatic macroinvertebrate assemblages, threatening the integrity of aquatic ecosystems in some parts of the country.^33^

Climate change is altering regional water cycles and intensifying weather extremes, leading to prolonged droughts, more frequent flooding, and extreme fire risk.^34^^,^^35^ The country has observed a notable increase in dry periods and a rise in the number of no-flow days in intermittent rivers.^36^ Perennial streams have also been impacted, with altered flow regimes resulting from periods of prolonged drought.^37^ However, the extensive remoteness of Australia, coupled with the sparse coverage of river gauges, complicates the effective management of surface water resources.^1^ These conditions highlight the pressing need for improved methodologies to monitor and understand surface water dynamics across Australia, and offer an ideal testing ground for the application of our methodology across varied environments.

#### Data sources

This study utilises five categories of data: (1) remote sensing-based surface water data, (2) river gauging-based stream discharge data, (3) river basin boundaries, (4) land cover, and (5) a Digital Elevation Model (DEM) for land surface topography. The spatial surface water extent, obtained from remote sensing, was derived from the Joint Research Center’s Global Surface Water Explorer (GSWE) dataset, accessible through Google Earth Engine.^20^^,^^38^ This cloud-based computational platform has been widely used for processing large-scale remote sensing data to understand surface water dynamics.^3^^,^^39^ The original GSWE dataset covers monthly data on the spatial water surface extent from March 1984 to December 2018 (418 months), which was used in this study. This raster-based data has a spatial resolution of 30 m, and each cell has one of the following three values to represent the surface water availability: 1 (water detected), 0 (no water), and NA (no data).

The river gauging-based discharge data was sourced from the Global Runoff Data Center (GRDC). The monthly calculated discharges from 371 river gauging stations across Australia were used. Observations indicating no flow (zero discharge) were excluded from our analysis.

River basin boundaries within Australia were delineated using the HydroBASINS dataset,^40^ which provides a hierarchically nested sub-basin structure across 12 levels following the Pfafstetter (PFAF) system of coding river basins. For this study, Level 8 was selected for its capacity to segregate river gauge stations into distinct basins, ensuring that most level-8 basins contained no more than one GRDC station. At this level, Australia was divided into 10,636 PFAF river basins, with an average area of 723.2 km^2^ per basin.

Land cover data was obtained from the Dynamic Land Cover Dataset (v2.1) provided by the Australian government. This dataset includes 22 land cover classes at 250-meter resolution, updated biennially from 2002 to 2015.^29^ The land cover map for the period from 1 January 2002 to 31 December 2003 was utilised, marking the midpoint of the dataset’s temporal range.

Topographic information for Australia was provided by the GEODATA 9 Second Digital Elevation Model (DEM-9S, Version 3), with a resolution of approximately 250 m (9 s).^30^ This is a regional dataset tailored to Australia. The 250 m resolution is sufficient for this research as the information was used only to calculate the coverage proportion of land cover types within each catchment.

### Quantification and statistical analysis

#### Data processing and variables

For data processing and preparation for analysis, GRDC stations were matched with corresponding PFAF basins. For each station-basin pair, only those months that had both river-gauging-based discharge data and remote-sensing-based data of spatial surface water extent were considered for further analysis. If pairs had fewer than three overlapping months of both data types, they were excluded to minimise the potential bias from missing data. Moreover, if over 10% of a PFAF basin lacked GSWE data in any given month, that basin-month combination was also excluded from the analysis.

The Relative Water Extent (RWE) was developed as the ratio of the Water Extent (WE) to the Maximum Water Extent (MWE), enabling a standardised comparison of surface water extent across different basins and time periods. It was calculated using Equation 1.

(Equation 1) $${RWE}_{b,m}=\frac{{WE}_{b,m}}{MWE_{b}}$$

RWE of river basin b in month m is equal to the area of WE of basin b in month m divided by the MWE of basin b during the data period (March 1984 to December 2018) (see Figures 1A and 1B). The Water Extent for each river basin in each month and the MWE for each river basin were computed using Google Earth Engine.

The effectiveness and accuracy of remote sensing data can be influenced by various factors, such as the availability and quality of satellite imagery^41^ and the physical characteristics of rivers.^16^^,^^42^^,^^43^ Land cover is also important as the real extent of spatial surface water coverage may be visually obscured by vegetation.^44^^,^^45^ In addition, urban areas can lead to misidentification of water bodies.^46^ Therefore, four categories of variables were constructed to represent these influencing factors: (1) *Data quality*, which was represented by the number of observations, or the month with both the river-gauge-based discharge and remote-sensing-based surface water extent data; (2) *Physical characteristics of river basins*, which were described by area (km^2^), mean altitude (m), altitude range (m), and Mean slope (%), derived from the Digital Elevation Model; (3) *Vegetation shading*, which was represented by a range of vegetation land cover types,^29^ including open Alpine grasses (%), open hummock grasses (%), open tussock grasses (%), closed tussock grasses (%), scattered shrubs and grasses (%), open shrubland (%), dense shrubland (%), open woodland (%), woodland (%), open forest (%), and closed forest (%); and (4) *Urbanisation*, which was quantified by the proportion of urban areas (%) within each river basin.^29^

#### Data analysis

Data analysis was conducted in two main steps: (1) to explore the relationships between river gauging-based and remote sensing-based data, and (2) to identify the factors influencing these relationships. Data analysis was conducted in Excel, R for Step 1, and SPSS for Step 2.

In the first step, we constructed linear and quadratic regression models to examine the relationship between the logarithms of river discharge (Q) and the RWE. Applying logarithmic transformations to both the independent and dependent variables reduced bias in the regression analysis. The coefficients were considered significant if the two-sided *p* value <0.05. The selection between linear and quadratic models was guided by the Akaike Information Criterion (AIC), which identified models with the best balance between explanatory power and simplicity. This step classified the relationship between river discharge and surface water extent in each basin into one of three categories.

(1) No significant relationship
(2) Linear relationship

(Equation 2) RWE=aQ+b

(3) Quadratic relationship

(Equation 3) $$RWE=aQ^{2}+bQ+c$$

In Step 2, two sets of binary logistic regression models were developed to identify: (1) the variables that predicted the presence of a significant relationship between river discharge and the spatial extent of surface water, irrespective of whether that relationship was linear or quadratic; and (2) the variables that determined whether a significant relationship was linear or quadratic. The forward selection likelihood ratio method was used for the variable selection process.

## References

[1] Krabbenhoft C.A., Allen G.H., Lin P., Godsey S.E., Allen D.C., Burrows R.M., DelVecchia A.G., Fritz K.M., Shanafield M., Burgin A.J. (2022). Assessing placement bias of the global river gauge network. Nat. Sustain..

[2] Gleason C., Durand M. (2020). Remote sensing of river discharge: A review and a framing for the discipline. Rem. Sens..

[3] Lin P., Feng D., Gleason C.J., Pan M., Brinkerhoff C.B., Yang X., Beck H.E., de Moraes Frasson R.P. (2023). Inversion of river discharge from remotely sensed river widths: A critical assessment at three-thousand global river gauges. Remote Sens. Environ..

[4] Alsdorf D.E., Rodríguez E., Lettenmaier D.P. (2007). Measuring surface water from space. Rev. Geophys..

[5] Sichangi A.W., Wang L., Yang K., Chen D., Wang Z., Li X., Zhou J., Liu W., Kuria D. (2016). Estimating continental river basin discharges using multiple remote sensing data sets. Remote Sens. Environ..

[6] Gleason C.J., Wang J. (2015). Theoretical basis for at-many-stations hydraulic geometry. Geophys. Res. Lett..

[7] Gleason C.J., Smith L.C. (2014). Toward global mapping of river discharge using satellite images and at-many-stations hydraulic geometry. Proc. Natl. Acad. Sci. USA.

[8] Birkinshaw S.J., O’Donnell G.M., Moore P., Kilsby C.G., Fowler H.J., Berry P.A.M. (2010). Using satellite altimetry data to augment flow estimation techniques on the Mekong River. Hydrol. Process..

[9] Prigent C., Jimenez C., Bousquet P. (2020). Satellite-derived global surface water extent and dynamics over the last 25 years (GIEMS-2). JGR. Atmospheres.

[10] Elmi O., Tourian M.J., Bárdossy A., Sneeuw N. (2021). Spaceborne River Discharge From a Nonparametric Stochastic Quantile Mapping Function. Water. Resour. Res..

[11] Tourian M.J., Schwatke C., Sneeuw N. (2017). River discharge estimation at daily resolution from satellite altimetry over an entire river basin. J. Hydrol. X..

[12] Durand M., Gleason C.J., Garambois P.A., Bjerklie D., Smith L.C., Roux H., Rodriguez E., Bates P.D., Pavelsky T.M., Monnier J. (2016). An intercomparison of remote sensing river discharge estimation algorithms from measurements of river height, width, and slope. Water Resour. Res..

[13] Gehring J., Duvvuri B., Beighley E. (2022). Deriving River Discharge Using Remotely Sensed Water Surface Characteristics and Satellite Altimetry in the Mississippi River Basin. Rem. Sens..

[14] Brakenridge G.R., Nghiem S.V., Anderson E., Mic R. (2007). Orbital microwave measurement of river discharge and ice status. Water Resour. Res..

[15] Tarpanelli A., Iodice F., Brocca L., Restano M., Benveniste J. (2020). River flow monitoring by sentinel-3 OLCI and MODIS: Comparison and combination. Rem. Sens..

[16] Huang Q., Long D., Du M., Zeng C., Qiao G., Li X., Hou A., Hong Y. (2018). Discharge estimation in high-mountain regions with improved methods using multisource remote sensing: A case study of the Upper Brahmaputra River. Remote Sens. Environ..

[17] Tetzlaff D., Carey S.K., McNamara J.P., Laudon H., Soulsby C. (2017). The essential value of long-term experimental data for hydrology and water management. Water Resour. Res..

[18] Shanafield M., Blanchette M., Daly E., Wells N., Burrows R.M., Korbel K., Rau G.C., Bourke S., Wakelin-King G., Holland A. (2024). Australian non-perennial rivers: Global lessons and research opportunities. J. Hydrol. X..

[19] Donchyts G., Baart F., Winsemius H., Gorelick N., Kwadijk J., van de Giesen N. (2016). Earth’s surface water change over the past 30 years. Nat. Clim. Change.

[20] Pekel J.F., Cottam A., Gorelick N., Belward A.S. (2016). High-resolution mapping of global surface water and its long-term changes. Nature.

[21] Sun S., Wang Y., Liu J., Cai H., Wu P., Geng Q., Xu L. (2016). Sustainability assessment of regional water resources under the DPSIR framework. J. Hydrol..

[22] Karthe D., Chalov S., Borchardt D. (2015). Water resources and their management in central Asia in the early twenty first century: status, challenges and future prospects. Environ. Earth Sci..

[23] Pedro-Monzonís M., Solera A., Ferrer J., Estrela T., Paredes-Arquiola J. (2015). A review of water scarcity and drought indexes in water resources planning and management. J. Hydrol..

[24] Damkjaer S., Taylor R. (2017). The measurement of water scarcity: Defining a meaningful indicator. Ambio.

[25] Omer A., Elagib N.A., Zhuguo M., Saleem F., Mohammed A. (2020). Water scarcity in the Yellow River Basin under future climate change and human activities. Sci. Total. Environ..

[26] Rentschler J., Salhab M., Jafino B.A. (2022). Flood exposure and poverty in 188 countries. Nat. Commun..

[27] Sharma S., Gomez M., Keller K., Nicholas R., Mejia A. (2021). Regional Flood Risk Projections under Climate Change. J. Hydrometeorol..

[28] Kundzewicz Z.W., Kanae S., Seneviratne S.I., Handmer J., Nicholls N., Peduzzi P., Mechler R., Bouwer L.M., Arnell N., Mach K. (2014). Le risque d’inondation et les perspectives de changement climatique mondial et régional. Hydrol. Sci. J..

[29] Australia Geoscience (2017). Dynamic Land Cover Dataset.

[30] Geoscience Australia (2008). GEODATA 9 Second Digital Elevation Data Version 3 and Flow Direction Grid 2008. https://researchdata.edu.au/geodata-9-second-grid-2008/3406854/.

[31] Kennard M.J., Pusey B.J., Olden J.D., MacKay S.J., Stein J.L., Marsh N. (2010). Classification of natural flow regimes in Australia to support environmental flow management. Freshw. Biol..

[32] Unmack P.J. (2001). Biogeography of Australian freshwater fishes. J. Biogeogr..

[33] Carey N., Chester E.T., Robson B.J. (2023). Loss of functionally important and regionally endemic species from streams forced into intermittency by global warming. Global Change Biol..

[34] Canadell J.G., Meyer C.P.M., Cook G.D., Dowdy A., Briggs P.R., Knauer J., Pepler A., Haverd V. (2021). Multi-decadal increase of forest burned area in Australia is linked to climate change. Nat. Commun..

[35] Head L., Adams M., Mcgregor H.V., Toole S. (2014). Climate change and Australia. Wiley Interdiscip. Rev. Clim. Chang..

[36] Sauquet E., Shanafield M., Hammond J.C., Sefton C., Leigh C., Datry T. (2021). Classification and trends in intermittent river flow regimes in Australia, northwestern Europe and USA: A global perspective. J. Hydrol. X..

[37] Peterson T.J., Saft M., Peel M.C., John A. (2021). Watersheds may not recover from drought. Science.

[38] Gorelick N., Hancher M., Dixon M., Ilyushchenko S., Thau D., Moore R. (2017). Google Earth Engine: Planetary-scale geospatial analysis for everyone. Remote Sens. Environ..

[39] Riggs R.M., Allen G.H., David C.H., Lin P., Pan M., Yang X., Gleason C. (2022). RODEO: An algorithm and Google Earth Engine application for river discharge retrieval from Landsat. Environ. Model. Software.

[40] Lehner B., Grill G. (2013). Global river hydrography and network routing: baseline data and new approaches to study the world’s large river systems. Hydrol. Process..

[41] Huang C., Chen Y., Zhang S., Wu J. (2018). Detecting, Extracting, and Monitoring Surface Water From Space Using Optical Sensors: A Review. Rev. Geophys..

[42] Ogilvie A., Belaud G., Massuel S., Mulligan M., Le Goulven P., Calvez R. (2018). Surface water monitoring in small water bodies: Potential and limits of multi-sensor Landsat time series. Hydrol. Earth Syst. Sci..

[43] Hou J., van Dijk A.I., Beck H.E. (2020). Global satellite-based river gauging and the influence of river morphology on its application. Remote Sens. Environ..

[44] Du J., Kimball J.S., Galantowicz J., Kim S.B., Chan S.K., Reichle R., Jones L.A., Watts J.D. (2018). Assessing global surface water inundation dynamics using combined satellite information from SMAP, AMSR2 and Landsat. Remote Sens. Environ..

[45] Revilla-Romero B., Thielen J., Salamon P., De Groeve T., Brakenridge G.R. (2014). Evaluation of the satellite-based global flood Detection System for measuring river discharge: Influence of local factors. Hydrol. Earth Syst. Sci..

[46] Chen F., Chen X., Van de Voorde T., Roberts D., Jiang H., Xu W. (2020). Open water detection in urban environments using high spatial resolution remote sensing imagery. Remote Sens. Environ..

